# Development of an Evidence-Based Best Practice Model for Teams Managing Crisis in Dementia: Protocol for a Qualitative Study

**DOI:** 10.2196/14781

**Published:** 2021-01-27

**Authors:** Miriam Stanyon, Amy Streater, Donna Maria Coleston-Shields, Jennifer Yates, David Challis, Tom Dening, Juanita Hoe, Brynmor Lloyd-Evans, Shirley Mitchell, Esme Moniz-Cook, Fiona Poland, David Prothero, Martin Orrell

**Affiliations:** 1 Division of Psychiatry and Applied Psychology School of Medicine University of Nottingham Nottingham United Kingdom; 2 Research and Development North East London Foundation Trust London United Kingdom; 3 Division of Nursing School of Health Sciences University of London London United Kingdom; 4 Division of Psychiatry University College London London United Kingdom; 5 Research and Innovation Nottinghamshire Healthcare NHS Foundation Trust Nottingham United Kingdom; 6 Faculty of Health Sciences University of Hull Hull United Kingdom; 7 School of Health Sciences University of East Anglia Norwich United Kingdom

**Keywords:** dementia, caregivers, crisis, mental health, home management

## Abstract

**Background:**

Teams working in the community to manage crisis in dementia currently exist, but with widely varying models of practice, it is difficult to determine the effectiveness of such teams.

**Objective:**

The aim of this study is to develop a “best practice model” for dementia services managing crisis, as well as a set of resources to help teams implement this model to measure and improve practice delivery. These will be the best practice tool and toolkit to be utilized by teams to improve the effectiveness of crisis teams working with older people with dementia and their caregivers. This paper describes the protocol for a prospective study using qualitative methods to establish an understanding of the current practice to develop a “best practice model.”

**Methods:**

Participants (people with dementia, caregivers, staff members, and stakeholders) from a variety of geographical areas, with a broad experience of crisis and noncrisis work, will be purposively selected to participate in qualitative approaches including interviews, focus groups, a consensus workshop, and development and field testing of both the best practice tool and toolkit.

**Results:**

Data were collected between October 2016 and August 2018. Thematic analysis will be utilized to establish the current working of teams managing crisis in dementia in order to draw together elements of the best practice.

**Conclusions:**

This is the first study to systematically explore the requirements needed to fulfill effective and appropriate home management for people with dementia and their caregivers at the time of mental health crisis, as delivered by teams managing crisis in dementia. This systematic approach to development will support greater acceptability and validity of the best practice tool and toolkit and lay the foundation for a large scale trial with teams managing crisis in dementia across England to investigate the effects on practice and impact on service provision, as well as the associated experiences of people with dementia and their caregivers.

**International Registered Report Identifier (IRRID):**

RR1-10.2196/14781

## Introduction

### Background

Home-orientated care is a key objective in the UK Dementia Strategy [1] to help people with dementia maintain their independence; however, fluctuations in health and the social needs of people with dementia and/or their caregivers can result in a breakdown of the caring process, making it difficult for the person with dementia to remain at home. This can lead to a crisis where the person with dementia may have to be admitted to an inpatient setting unless skilled management of the situation within the community can be employed. While support from community mental health teams or specialist dementia services exists, waiting times and indirect care pathways can make access difficult [2] at a critical time when the person with dementia may be experiencing an increase in behavioral and psychological symptoms. A specialist rapid access intervention service for people with dementia and caregivers facing crisis could be an effective support mechanism to prevent the breakdown in care at home.

Avoidance of crisis in dementia could reduce unnecessary emergency hospital admissions. An Alzheimer Society report found that one in 10 respondents sought hospital admission for their relative owing to lack of access to community support [3]. Community support for people of working age is well specified, with identified teams (often called crisis resolution teams) in place to avoid hospital admission, but a national survey found that only 16% of these general adult crisis teams accept people with dementia onto their caseload [4]. The availability of teams specific for older people with dementia is limited and variable, with differences in both the remits and names of older adult teams. The same survey identified only 30 standalone dementia or older adult crisis teams nationwide. A subsequent online scoping survey of 62 managers of teams managing crisis in dementia identified wide variations in care pathways and types of services managing crisis in dementia. Such services include dementia intensive support teams, mental health intensive recovery teams, dementia crisis support teams, dementia rapid response teams, and intensive recovery intervention services [5]. This survey further identified variations in how services have developed and in teams’ objectives in either preventing events that can lead to a breakdown in care or dealing with the aftermath of care breakdown and preventing hospital admission. Regardless of whether a team has a preventative or management remit, services typically aim for cost saving by maintaining the ability of the person with dementia to stay at home. In many areas, however, services are unavailable and hospital admissions are unavoidable.

A study of nine focus groups found that people with dementia, caregivers, and staff value a coordinated evidenced-based approach to crisis avoidance, one which takes into account increasing dementia symptoms, caregiver inability to continue to provide care, deteriorating physical health of the person with dementia or family caregiver, unsuitability of the home environment, and insufficient community services [6]. With this in mind and drawing on findings from the scoping survey described above [5], research proposed in this protocol, which is part of the Achieving Quality and Effectiveness in Dementia Using Crisis Teams (AQUEDUCT) program funded by the National Institute for Health Research (NIHR) under its Program Grants for Applied Research scheme (RP-PG-0612-20004), will lead to the development of a “best practice model” specific for teams managing crisis in dementia. This is necessary given the current lack of evidence to support, develop, and promote such services.

### Aims

The research described in this protocol aims to explore current practice and to develop an intervention (the best practice tool and toolkit) by addressing the following questions: (1) What is the current practice for a team managing crisis in dementia? (2) What is considered the “best practice” for a team managing crisis in dementia? (3) By which standards should the practice of a team managing crisis in dementia be measured? (4) What does a team managing crisis in dementia require to improve its practice?

## Methods

### AQUEDUCT Research Program

The AQUEDUCT research program is comprised of the following three work packages (WPs): work package 1 (WP1), which concerns the development of the intervention (the best practice tool and toolkit); work package 2 (WP2), which involves a feasibility study for this intervention; and work package 3 (WP3), which involves a full trial of the intervention. This paper describes the protocol for WP1 only.

The research in this WP is informed by the process for promoting service improvement developed by the US evidence-based practice (EBP) program, which has demonstrated effectiveness in supporting high-fidelity implementation of a range of complex interventions and service models [7]. Components of the EBP approach include [8,9] (1) defining a model of best practice with detailed specification; (2) developing a means of assessing adherence to this model; and (3) developing a package of implementation resources to support service improvement and greater adherence to the “best practice model.”

The intervention to be developed, consisting of the best practice tool and toolkit, will be generated through an iterative understanding of current practice and what is considered to be the best practice in teams managing crisis in dementia. This work will build upon previously conducted research, namely the “Support at Home–Interventions to Enhance Life in Dementia” (SHIELD) program [10]. This previous study developed the home treatment package (HTP), a tool for assessing people with dementia and their caregivers at times of crisis. It incorporates a number of components, including the threshold assessment grid (TAG) risk assessment [11], the Camberwell assessment of needs in the elderly (CANE) [12], a care planning template, a discharge planning template, exemplary case studies, and an advisory protocol. The HTP will be revised in phase 3 of WP1, as described below.

### Sample

Sample sizes to be used in each phase of this research are similar to those considered sufficient to achieve data saturation in previously conducted research on older people and working age crisis management [6,13].

### Inclusion Criteria

For this research, we will consider inclusion of (1) staff members of teams managing crisis in dementia who have been employed by the team managing crisis in dementia for a minimum of 6 months and have been working directly with people with dementia; (2) people with dementia who have a diagnosis or probable diagnosis of dementia, have been discharged from the team managing crisis in dementia within the past 6 months, have the mental capacity to provide informed consent, and have some recollection of the involvement of the team managing crisis in dementia in their care; and (3) caregivers who have cared for people with a diagnosis or probable diagnosis of dementia who received input from the team managing crisis in dementia within the past 6 months.

### Recruitment and Consent

Several different groups of participants will be involved in this research, and they will be recruited in various ways. National Health Service (NHS) Trusts across England will be approached initially by a member of the AQUEDUCT research team who will explain the study to research and development contacts, so that the assessment of the capability and capacity to complete the research can be initiated. NHS Trusts that include appropriate teams will then ascertain capability and capacity for involvement in individual phases of the research (rather than involvement in the protocol as a whole) by discussing the research with team managers. Once the team manager has agreed to their team’s involvement in that phase of the protocol, individual staff members from the widest possible range of roles and bandings will be approached by the manager to discuss participation in the study.

People with dementia, caregivers, and other staff members who work with the teams (stakeholders) will be approached initially by a member of the clinical team and asked if they would be willing to speak with a member of the AQUEDUCT research team. If they are interested in participating, they will then receive a copy of the relevant information sheet. A patient and public involvement (PPI)-approved dementia-friendly participant information sheet will be created by the research team to facilitate understanding of the research for people with dementia. Potential participants will be given up to 3 days to decide whether to participate, after which point, if they indicate to a member of the clinical team that they are happy to participate, the AQUEDUCT research team member will answer any questions and discuss the time and location of the interview or focus group.

For the consensus workshop, participants will be purposively recruited based on their previous contact with a team managing crisis in dementia and their willingness to be recontacted regarding future AQUEDUCT research activity. A personal invitation will be sent to them with all information and details about the workshop. They will be asked to return an expression of interest to indicate their intention to attend, and consent will be taken by members of the AQUEDUCT research team on the day of the workshop itself.

Prior to all research activities, a member of the AQUEDUCT research team will work through the relevant information sheet with the prospective participant, offer an opportunity for further questions, and take written consent. On signing the consent form, all participants will be allocated a unique identification number to ensure their anonymity during analysis and reporting of findings.

### Patient and Public Involvement

The AQUEDUCT program overall will actively engage with people with dementia and caregivers as PPI representatives. The role of PPI will be incorporated into all stages of the research, including advising on study documentation and participant recruitment procedures, assisting in data collection as coresearchers, commenting on the suitability of data analysis, and taking part in the AQUEDUCT Program Steering Group and AQUEDUCT PPI Reference Group. In this way, every stage of the research process will be informed by service user experience and expertise, and the research will adhere to its objectives of benefitting people with dementia, caregivers, and members of the public.

### Phase 1: Mapping Current Practice and Identifying the Best Practice

**Figure 1:**
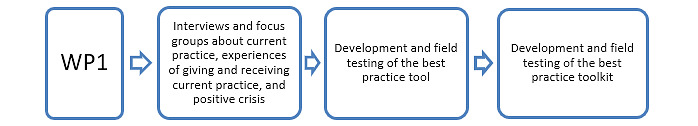
Design and three constituent phases of work package 1 (WP1).

This is a prospective study using qualitative methods to garner a broad understanding of the necessary elements of service provision for effective crisis management and resolution for people with dementia and their caregivers. The design and three constituent phases of WP1 are illustrated in Figure 1.

In the first phase, interviews will be carried out (following purposive sampling) to garner the perspectives of people with dementia, caregivers, staff members of teams managing crisis in dementia, and individuals who work with teams managing crisis in dementia (stakeholders) on current practice and the experience of providing or receiving care. Participants who have accessed, had contact with, or worked in a team managing crisis in dementia are considered eligible for interview. Interviews will map the scope of a service and will document team composition, geographical characteristics and practical operation, links with other services, communication and decision-making, and service evaluation processes. The interviews will result in collection of information concerning clinical processes and procedures, together with information on what works well and what does not work well in order to consider the best practice and possible facilitators of the best practice (see Multimedia Appendix 1 for the interview schedule being used).

Semistructured focus groups will then be carried out with people with dementia, caregivers, staff members of teams managing crisis in dementia, and individuals who work with teams managing crisis in dementia to consider the best practice further and to identify what facilitates the positive working of teams managing crisis in dementia. These groups will ascertain the setup of teams managing crisis in dementia, barriers and facilitators to positive working, and examples of good practice. Data will be analyzed using thematic analysis to develop items for the “best practice model.”

Sixty participants from five teams managing crisis in dementia will be recruited for individual interviews. AQUEDUCT team researchers and PPI coresearchers will then facilitate nine focus groups of four to six participants each.

Incorporating two different stages of qualitative exploration (individual interviews and focus groups) will be the most appropriate methodology to enable understanding of the current practice and identification of the best practice, as it will provide an opportunity to elicit both individual and context-specific characteristics [14]. Through this iterative process, it will be possible to recognize key characteristics in dementia crisis team working in order to increase the validity and richness of the findings.

### Phase 2: Development and Field Testing of the Best Practice Tool

A best practice tool will be created to be used by teams managing crisis in dementia to measure their current practice and the extent to which they fit the “best practice model.” The process of testing the best practice tool will be derived from the CORE study procedure, a NIHR-funded program that developed a fidelity scale of best practice for working-age crisis resolution teams [15]. The development of the best practice tool detailed in this paper aligns with the development of the CORE Fidelity Scale.

EBP principles [8,9] will be used to draw together evidence gathered during the qualitative phase of this protocol to form a “best practice model” and to develop the first iteration of the best practice tool. A consensus workshop will revise and validate the best practice tool to create the next version. The workshop will involve at least 25 attendees, including PPI representatives, staff and managers of teams managing crisis in dementia, NHS staff from primary and secondary care who interface with teams managing crisis in dementia, senior trust managers, commissioners, and academics.

The revised version of the best practice tool will then be field tested with 12 teams managing crisis in dementia and five older peoples’ community mental health teams that do not have a dedicated dementia crisis response function, to establish face and content validity. Comparing best practice tool scores for these two types of teams will provide construct validity, ensuring that the practice quality of crisis teams, rather than the practice quality of generic mental health teams, is measured by the best practice tool. Each item of the tool will specify various types of evidence that can be inspected to determine whether the team meets the scoring criteria and will be weighted so that the team will receive an overall best practice tool score out of 100.

Each team will take part in a review day during which three reviewers (a member of the AQUEDUCT research team, a PPI member, and a clinician who works with people with dementia) will rate the practice of the team according to the “best practice model.” Evidence will be collected from various sources, including interviews with team members, team managers, staff from other services who work closely with the teams, people with dementia, and caregivers; case note and paperwork reviews; and a visual check of the team base. Reviewers will compile and evaluate all data throughout the day to agree on a best practice tool score. The face and content validity of the best practice tool will be assessed using these data.

The best practice tool will enable identification of “gaps” in the current practice of each team managing crisis in dementia that can be filled by use of the toolkit, the development and implementation of which is outlined below.

### Phase 3: Development and Field Testing of the Best Practice Toolkit

The best practice toolkit will include the HTP developed during the SHIELD study, which has been mentioned above. A briefing will be carried out with two senior staff members of teams managing crisis in dementia to determine suitability for use and ease of completion of the HTP. The purpose of this is to modify the HTP where required before it is incorporated as an element of the toolkit. The briefing will involve one day of training for the staff members of teams managing crisis in dementia on the purpose and application of the HTP (the latter incorporating case study examples). Thereafter, staff members will draw on their own clinical experience to complete the HTP and provide feedback on the process of doing so to the AQUEDUCT research team.

Additional elements of the best practice toolkit will be determined by drawing on information generated from the qualitative work to identify elements of best practice, considering in particular how teams can best fulfill the criteria laid out in the “best practice model.” The toolkit will promote best practice through the use of templates and documents that can be mapped onto the best practice tool.

Five teams managing crisis in dementia will field test the best practice toolkit. Staff members from these teams managing crisis in dementia will receive online training in the use of the toolkit, and two AQUEDUCT team researchers will then visit these staff in their place of work to discuss their best practice tool score and areas for improvement. The staff members of teams managing crisis in dementia will agree with the AQUEDUCT team researchers about which elements of the toolkit they will implement over a period of 4 weeks, and after this time, they will provide feedback on the suitability of those elements for use in their team. During the 4-week period, an AQUEDUCT team researcher will conduct weekly telephone calls with the team managing crisis in dementia to record usage of the toolkit elements. Following receipt of all feedback from the five teams managing crisis in dementia, the toolkit will be amended for future use in the feasibility study.

### Governance

All information will be treated as confidential, with adherence to the NHS Code of Confidentiality [16], General Data Protection Regulation [17], and Good Clinical Practice guidelines [18]. All insurance and indemnity arrangements will be covered by the study sponsor, Nottinghamshire Healthcare NHS Foundation Trust. Ongoing progress of the research will be monitored by the Program Management Group comprised of the coapplicants who have been awarded the NIHR grant, and a Program Steering Group that will be independent of the sponsor and other interested parties, in association with the chief investigator and program manager, both of whom are consultant clinicians. Overall, this research will benefit from and be guided by experts from the fields of older adult psychiatry, clinical psychology, mental health nursing, social work, and occupational therapy owing to the clinical background and expertise of the coapplicants on the grant, as well as voluntary services for older people, PPI, and research methodology and dissemination.

## Results

Ethical approval was provided by the West Midlands (Black Country) Research Ethics Committee (reference: 16/WM/0273) on August 4, 2016, and Health Research Authority approval was provided on August 24, 2016. Data collection began in October 2016 and was completed by the end of August 2018.

Interview and focus group data will be analyzed using the six-phase thematic analysis method [19] to establish the current working of teams managing crisis in dementia and to draw together elements of best practice. This analysis process involves researchers (1) becoming familiar with the data, (2) generating initial codes from the data, (3) searching for themes, (4) reviewing themes, (5) defining and naming themes, and (6) writing up the thematic analysis. Data will be organized using the framework method [20] to assist in comparison of data within themes and across participants. Results will inform items for the evidence-based “best practice model,” and the first version of the best practice tool will be developed using evidence-based practice principles [21]. Feedback data from reviewers, teams, and others who take part in the review days will then be used to evaluate the usability and feasibility of the best practice tool, and the outcome data from this version will be compared between teams managing crisis in dementia and noncrisis teams to ensure that the best practice tool is specific to teams managing crisis in dementia as opposed to generic older adult mental health teams.

## Discussion

This protocol presents extensive qualitative methods and an iterative approach to enable accumulation of in-depth knowledge about the characteristics, processes, and policies of the working of teams managing crisis in dementia. The use of both semistructured interviews and focus groups will mean that decision-making processes and rationales given for ways of working can be explored fully [14] and incorporated into an evidence-based “best practice model.”

Integral to this research is the role of people with dementia and their caregivers, ensuring that their views and experiences will be incorporated. In particular, it is proposed that interviews and focus groups will be carried out with people with dementia, so that this research does not assume what constitutes best practice on their behalf. PPI and research cocreation are built into this protocol to reduce the possibility of this research becoming detached from the working of teams managing crisis in dementia in practice, as experienced by those who receive it.

As this work will make use of participants’ retrospective accounts of the involvement of teams managing crisis in dementia, it is possible that in the case of people with dementia, memory difficulties may lead to gaps in details provided. Caregiver interviews will also be included to compensate for this; however, as general impressions from people with dementia (to include their residual emotions generated by input from teams managing crisis in dementia) are considered crucial to the development of a “best practice model” that takes into account “softer” aspects of care, such information will be collected whenever possible

The research outlined in this protocol will result in the development of a “best practice model” for teams managing crisis in dementia and a best practice tool by which fidelity to this model can be measured. To date, no such model exists. Additionally, dementia crisis working has been inconsistent and the quality of care delivered has been variable and difficult to measure owing to the lack of quality indicators and lack of standardization across services. This research has the potential to address this current variability in practice. Such variability may be in part due to the variety of team models existing at present, ranging from those that are dementia specific to those serving older people generally (and which thus have a remit for functional disorders) and to those serving adults of all ages who are experiencing a mental health crisis. The “best practice model” will be promoted through the best practice tool and toolkit that will provide teams with a benchmark by which to measure their practice specific to dementia working and the resources required to improve delivery of their practice, respectively. Ultimately, it is expected that the best practice tool will be suitable for self-completion by the staff of teams managing crisis in dementia who will then be able to identify their own practice areas requiring improvement. This approach will be trialed in a subsequent feasibility study. As the “model of best practice” is based almost exclusively on stakeholder opinion rather than on objective empirical evidence, its validity must be confirmed by establishing the relationship between good model adherence and better outcomes. This will be explored in a future large-scale trial.

## Appendix

Multimedia Appendix 1 Achieving Quality and Effectiveness in Dementia Using Crisis Teams (AQUEDUCT) work package 1 interview guides.

## Abbreviations

AQUEDUCT: Achieving Quality and Effectiveness in Dementia Using Crisis Teams
EBP: evidence-based practice
HTTP: home treatment package
NHS: National Health Service
NIHR: National Institute for Health Research
PPI: patient and public involvement
SHIELD: Support at Home–Interventions to Enhance Life in Dementia
WP: work package
