# Supplementary material for: Development of an Evidence-Based Best Practice Model for Teams Managing Crisis in Dementia: Protocol for a Qualitative Study
Source: JMIR Res Protoc. 2021 Jan 27;10(1):e14781. doi: 10.2196/14781 (PMC7875693; doi:10.2196/14781)
Supplement: Multimedia Appendix 1 [file resprot_v10i1e14781_app1.docx]

**Interview Guide for Work Package 1.1.1 – Interviews With TMCD Staff Members About Current Practice**

- Aim of the project
- Aim of the interview (introduce all the topics)
- Consent for recording
- Personal details e.g. job role, time in post

1. I’m going to ask you some questions about the service your team provides.

2. I’d now like to ask you about the characteristics of your team.

3. I’d like to talk to you now about where your service is located.

4. Now I’d like to ask you about the team’s links with other services such as social care and primary care.

5. I’d like to ask you a bit about how your team communicates.

6. I’d like to ask you about the decision making processes in your team.

7. I’d like to ask about how your team measures clinical outputs.

8. Now I’d like to ask you about your team’s reflexivity and innovation. What I mean by that is about how your team comes together to think about processes, service development and how you can implement new ideas.

9. Is there anything else you’d like to say about your team and how it works that we haven’t already covered?

10. Thank you!

**Interview Guide for Work Package 1.1.2 – Interviews With TMCD Staff Members, Service Users and Carers About the Experience of Giving and Receiving Current Practice**

**Service User**

• Aim of the project

• Aim of the interview (introduce all the topics)

• Consent for recording

• Personal details e.g. Could you just tell me about your living circumstances at the moment e.g. do you live by yourself? Does someone come in to help you? What kind of help do you receive on a regular basis?

1. **What brought you into contact with (team)?**
2. **What happened next?**
3. **How did the team talk to you about ending their period of care?**
4. **Was there anything about you involvement with the team that you thought was really positive?**
5. **Was there anything about you involvement with the team that you thought was less positive?**
6. **Imagine you were looking after a friend who was experiencing a crisis like this, what would you like to happen?**
7. **Further comments?**
8. **Thanks!**

**Carers**

- Aim of the project

• Aim of the interview (introduce all the topics)

• Consent for recording

• Personal details e.g. Could you just tell me about how you look after ____ e.g. do you live with them? What kind of help do you give? How often?

1. **What brought you into contact with (team)?**
2. **What happened next?**
3. **How did the team talk to you about ending care of the person you care for?**
4. **Was there anything about you involvement with the team that you thought was really positive?**
5. **Was there anything about your involvement with the team that you thought was less positive?**
6. **If you were a person with dementia experiencing a crisis, how would you like people to treat you?**
7. **If you were in control of a crisis team, what changes would you make?**
8. **Further comments?**
9. **Thanks!**

**Staff members**

• Aim of the project

• Aim of the interview (introduce all the topics)

• Consent for recording

• Personal details e.g. job role, time in post

- How is it that you came to work for the crisis team?
  - Ownership of the job e.g. moulding the job to what you want it to be

1. **Thinking about how your team provides care, can you think of a case where you were really satisfied with how your team provided care and explain what happened?**
2. **Can you think of a case where you were less happy with the care that was provided by the team or things didn’t work out how you would have wished? Can you explain what happened?**
3. **What helps you provide ‘ideal practice’?**
4. **Can you think of things that get in the way of ‘ideal practice’?**
5. **How do you feel when things go well, less well?**
6. **How do you think current practice can be improved?**
7. **Further comments?**
8. **Thanks!**

**Questions for Focus groups Work Package 1.1.3 – Focus Groups on Positive TMCD Practice**

• Aim of the project

• Aim of the interview (introduce all the topics) + task

• Consent for recording

• Personal details e.g. job role, time in post

1. **Now we’ve introduced ourselves the first thing I would like to do is agree on a definition of crisis for people with dementia. So far in this project we have defined a crisis as ‘an urgent need for an assessment and intervention for a person living in the community’. What do you think of this/does your team have a definition? So what kind of crises might there be for people with dementia in the community?**
2. **Post-it notes exercise to describe best practice of crisis teams (in pairs)**
3. **So if we have a team that is seeking to resolve crisis for people with dementia, if we were to start from scratch, what would that service look like?**
4. **Feedback to the group**

**What’s your top 5 most important**

1. **What do you think are the potential barriers to being able to practice in this way?**
2. **What would facilitate this kind of practice?**
3. **Is there anything else you would like to say about ‘best practice’ in a crisis team?**
